# Unlocking Histatin Potential against *Candida albicans* and *Streptococcus mutans* Biofilms: Targeting the Extracellular Matrix While Preserving Oral Cell Integrity

**DOI:** 10.1021/acsomega.5c02108

**Published:** 2025-06-18

**Authors:** Luana Mendonça Dias, Lina M. Marin, Ana Claudia Pavarina, Walter L. Siqueira

**Affiliations:** 1 College of Dentistry, 7235 University of Saskatchewan (USASK), 105 Wiggins Road, Saskatoon, SK S7N 5E4, Canada; 2 Laboratory of Applied Microbiology Department of Dental Materials and Prosthodontics, Universidade Estadual Paulista “Júlio de Mesquita Filho”, Faculdade de Odontologia de Araraquara, Rua Humaitá, 1680, Araraquara, SP 14803-901, Brazil

## Abstract

Denture stomatitis affects up to 60% of prosthesis users due to biofilms mainly composed of *Candida albicans* and *Streptococcus mutans*, which persist the treatments because of their resilient extracellular matrix (ECM). This study tested four proteins/peptides—histatin 3 (His3), histatin 5 (His5), DR9-RR14, and RR14—on these mixed biofilms grown on acrylic resin. Using previously determined biofilm inhibitory concentrations (BIC-2), their effects on biofilm viability, ECM components (proteins, extracellular DNA, and polysaccharides), and biofilm structure were assessed. His3 and His5 were the most effective, reducing biofilm cells by 46 and 41% and significantly decreasing ECM components. DR9-RR14 and RR14 had moderate effects. Imaging confirmed that His3 and His5 disrupted the biofilm structure. Cytotoxicity tests showed that all proteins/peptides were safe for gingival fibroblasts. Among the proteins/peptides evaluated, His3 showed the highest effectiveness, making it a promising candidate for preventing biofilm formation and ECM maturation, suggesting its potential use in treating denture stomatitis.

## Graphical abstract

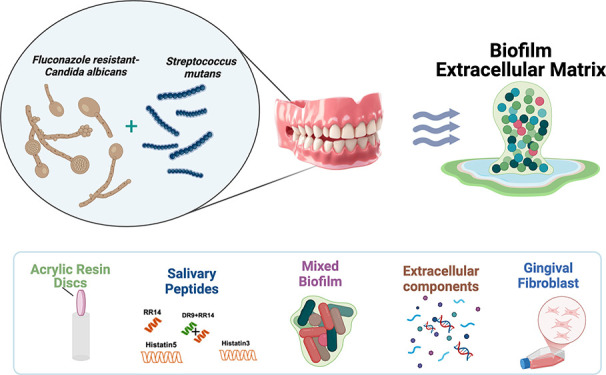

## Introduction

*Candida albicans* is a commensal fungus present in the oral microbiota of healthy individuals.^1^ In patients who wear acrylic dentures, the colonization of this fungus can progress to an inflammatory reaction known as “denture stomatitis”,^1^ which is common among 60% of prosthesis users.^2^ Although *C. albicans* is the main pathogen of denture stomatitis, bacteria such as *Streptococcus mutans* have also been found in biofilms formed on acrylic dentures^3^ *C. albicans* and *S. mutans* may interact in synergistic manners. For instance, *C. albicans* is not able to efficiently metabolize sucrose, but it benefits from free glucose and fructose generated from the metabolism of sucrose by *S. mutans*. Additionally, in the presence of sucrose, *S. mutans* synthesizes exopolysaccharides (EPS), a component of the extracellular matrix (ECM) that promotes strong bonds among different microorganisms resulting in the formation and cohesion of biofilm.^4^

Biofilms are organized, structured, and functional biological communities, consisting of a clustered cell surrounded by a polymeric ECM.^5^ The ECM structure is composed of polysaccharides, proteins, and nucleic acids; they contribute to the protection of the biofilm and facilitate stable interactions among cells.^5,6^ Furthermore, the presence of ECM not only delays infection but also contributes to an increased resistance of pathogenic biofilms to antimicrobial agents.^6^ Current conventional therapies for fungal infections face significant challenges, including antimicrobial resistance and a limited spectrum of activity.^7^ To overcome this limitation, new therapeutic approaches are needed to control the growth of these microorganisms on the oral mucosa and the acrylic surfaces of removable prostheses.

The successful use of antimicrobial proteins/peptides (AMPs) in treating infections, particularly those caused by biofilm-related and multidrug-resistant microorganisms, has encouraged scientists to explore new methodologies for improving the synthesis, isolation, purification, analysis, and quantification of these compounds.^8^ AMPs also have innate characteristics of protecting the immune system against infection by pathogens, including viruses, bacteria, fungi, and parasites.^8,9^ Promising proteins/peptides and proteins have been found in the acquired enamel pellicle (AEP), the salivary film formed on dental enamel,^10–12^ with some AMPs originating from salivary proteolysis, such as the proteins histatin and statherin.^10–12^ Notably, histatins have the capacity to inhibit *C. albicans*,^4,13^ and they also impact mitochondrial swelling in the Krebs cycle, resulting in a reduction of adenosine triphosphate production.^14^ Additionally, histatins also reduce the adhesion of *S. mutans*, the most cariogenic microorganisms in dental biofilm, to hydroxyapatite.^15^ However, studies exploring the action of histatin 3 (His3) and histatin 5 (His5) against *C. albicans* and *S. mutans* have been limited to single-species biofilms.

Hybrid peptides engineered from salivary proteins were also developed from the biological domains of statherin and histatin.^16,17^ For instance, the peptides DR9, located in the N-terminal portion of the native protein statherin, also inhibits the formation of *C. albicans* hyphae, reducing their virulence and cell proliferation when compared to statherin.^18^ RR14 is derived from the native protein histatin 3 ^16^ and has an effect against the biomass of *S. mutans* biofilms biomass.^15^

There are no studies in the literature that tested the antimicrobial effect of DR9-RR14, RR14, His3, and His5 on mixed biofilm composed of *C. albicans* and *S. mutans*. Moreover, it is not possible to ensure safe use of these proteins/peptides as an antimicrobial therapy without concomitantly evaluating their biocompatibility in human oral cells. Thus, the aim of the present study was to investigate the effect of the peptides DR9-RR14 and RR14, and proteins, His3 and His5 against fluconazole-resistant *C. albicans* and *S. mutans* mixed biofilm formed under acrylic resin and their ECM, as well as to assess their cytotoxicity on human gingival fibroblasts (FGH).

## Results

### Biofilm Inhibitory Concentration (BIC-2) by Absorbance and CFU/mL

A concentration of 256 μM His3 led to a 57% reduction in mixed biofilm absorbance (optical density: OD) compared to the growth control (GC) (Figure 1a). Likewise, the 512 μM concentration of His5 resulted in a 62% reduction (Figure 1b). In addition, DR9-RR14 at a concentration of 2048 μM reduced mixed biofilm absorbance by 50% (Figure 1c), while RR14 at 4096 μM showed a reduction of 53% compared to that of the GC (Figure 1d).

**Figure 1:**
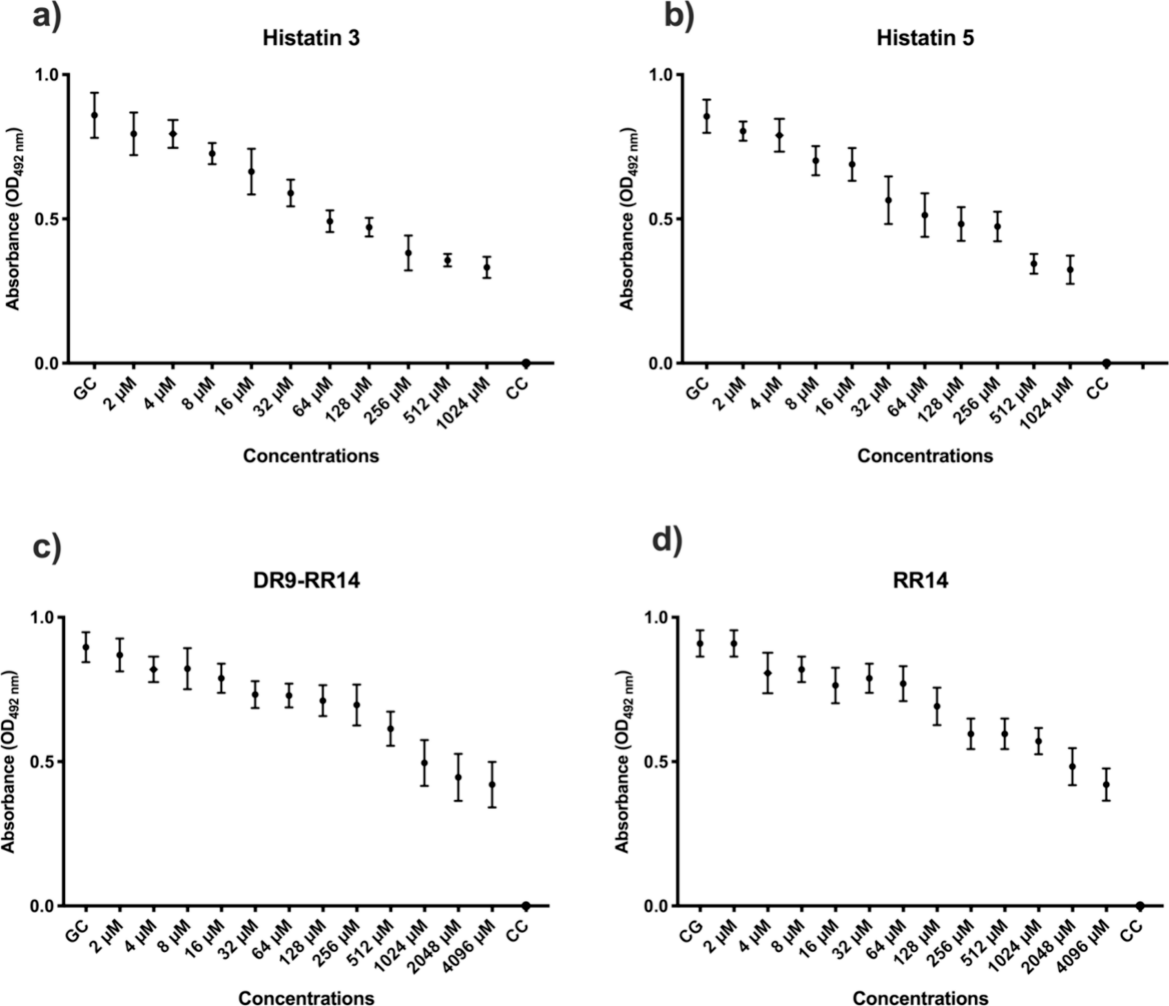
Median and 95% confidence interval of BIC-2 measured by absorbance OD 492 nm of mixed biofilm of *Candida albicans* and *Streptococcus mutans* against proteins (a) histatin 3 and (b) histatin 5 at predetermined concentrations: 2, 4, 8, 16, 32, 64, 128, 256, 512, 1024, 2048, and 4096 μM. Peptides (c) DR9-RR14 and (d) RR14 at predetermined concentrations: 2, 4, 8, 16, 32, 64, 128, 256, 512, and 1024 μM. CC: contamination control (vehicle only). GC: growth control (without treatment). Points: data medians. Error bars: minimum and maximum values. The non-intersection of error bars indicates statistical significance (*p* < 0.05), based on 95% confidence intervals (*n* = 9/group).

Regarding CFU/mL quantification, it was observed that 256 μM His3 reduced CFU/mL by 59% compared to the GC (Figure 2a) and 512 μM His5 led to a reduction of 62% (Figure 2b). Similarly, DR9-RR14 and RR14 at 2048 μM decreased the CFU/mL mixed biofilm by 50% (Figure 2c,d).

**Figure 2:**
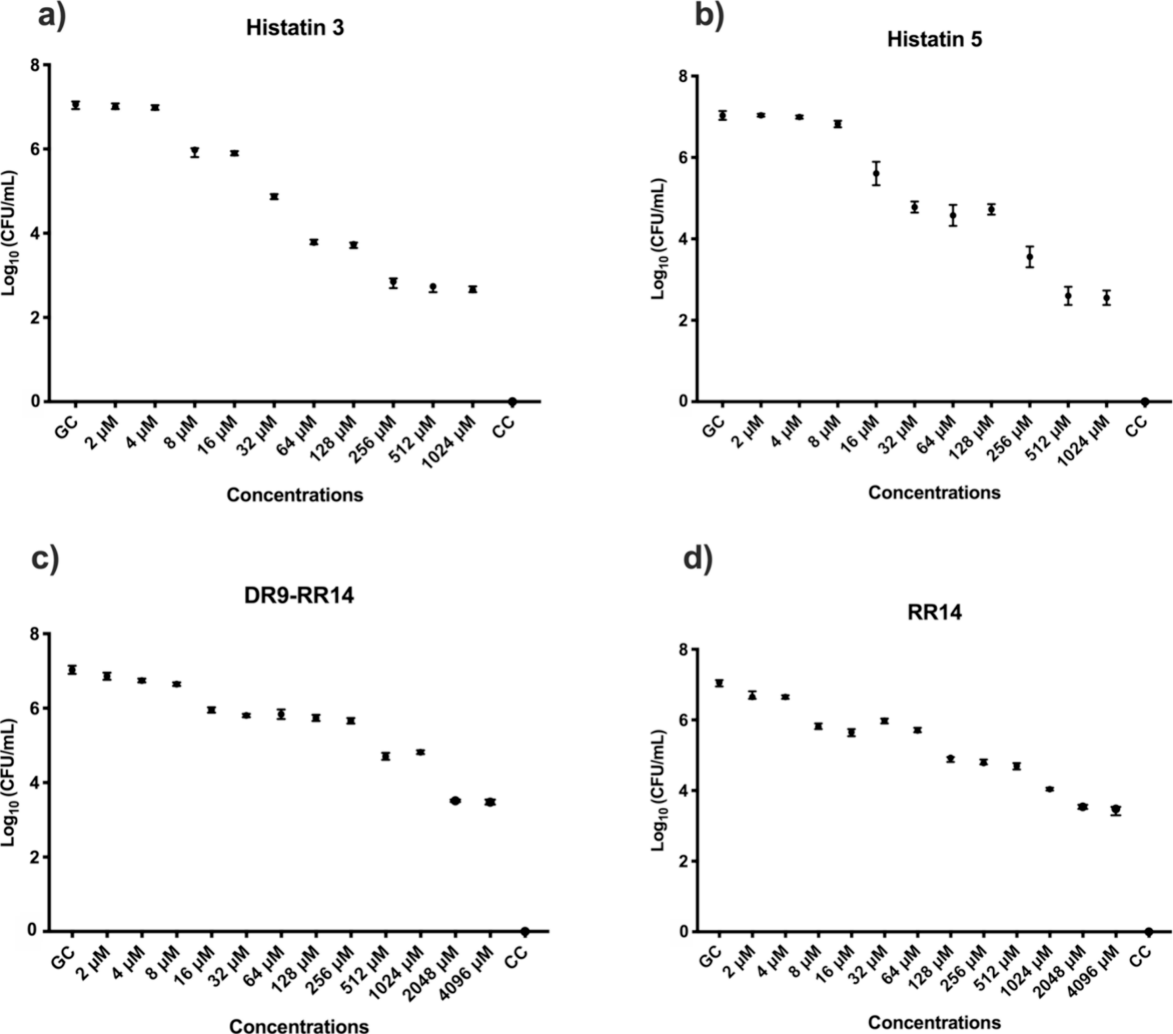
Median and 95% confidence interval of BIC-2 measured by CFU/mL of mixed biofilm of *C. albicans* and *S. mutans* against proteins (a) histatin 3 and (b) histatin 5 at predetermined concentrations: 2, 4, 8, 16, 32, 64, 128, 256, 512, 1024, 2048, and 4096 μM. Peptides (c) DR9-RR14 and (d) RR14 at predetermined concentrations: 2, 4, 8, 16, 32, 64, 128, 256, 512, and 1024 μM. CC: contamination control (vehicle only). GC: growth control (without treatment). Points: data medians. Error bars: minimum and maximum values. The non-intersection of error bars indicates statistical significance (*p* < 0.05), based on 95% confidence intervals (*n* = 9/group).

### Effect of Peptides/Proteins on Mixed Biofilm Viability by CFU/mm^2^

The viability of cells (CFU/mm^2^) in mixed biofilms of *C. albicans* and *S. mutans* growth on acrylic resin was significantly reduced after the treatment with proteins/proteins (Figure 3a). His3 at a concentration of 256 μM reduced the colony count (CFU/mm^2^) viability by 46% compared to the NaCl group (*p* = 0.003), representing the greatest reduction observed. When analyzed individually within the mixed biofilm, *C. albicans* showed a 60% reduction in CFU/mm^2^, while that of *S. mutans* was reduced by 40% (Figure 3a). Similarly, His5 at 512 μM also significantly decreased the viability (CFU/mm^2^) of the mixed biofilm by 46% (*p* = 0.004) compared to the NaCl group. The mixed biofilm exhibited a reduction of 57 and 43% in CFU/mm^2^, for *C. albicans* and *S. mutans*, respectively (Figure 3b).

**Figure 3:**
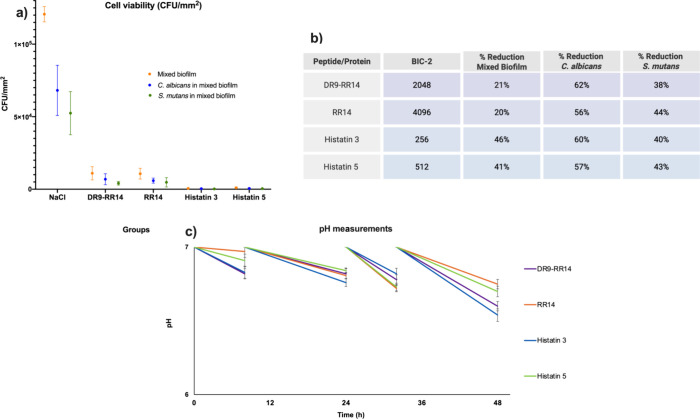
(a) Median and 95% confidence interval of mixed biofilm (orange color: *Candida albicans* and *Streptococcus mutans* formed on acrylic resin), *C. albicans* in mixed biofilm (blue color), and *S. mutans* in mixed biofilm (green color) against proteins/peptides DR9-RR14 (2048 μM) and RR14 (4096 μM), and protein histatin 3 (256 μM) and histatin 5 (512 μM); NaCl: experimental control. Points: data means. Error bars: minimum and maximum values. The nonintersection of error bars denotes statistical difference according to the 95% confidence interval (*p* < 0.05). (b) Table with reductions (%) of the mixed biofilm formed on acrylic resin against peptides (DR9-RR14; RR14; histatin 3; histatin 5; and reductions of *C. albicans* and *S. mutans* in mixed biofilm (*p* < 0.05) (*n* = 9/group). (c) pH measurements after 0, 12, 24, 36, and 48 h of the proteins/peptides DR-RR14, RR14, histatin 3, and histatin 5.

DR9-RR14, applied at a concentration of 2048 μM, reduced the viability of the mixed biofilm (CFU/mm^2^) by 21% (*p* = 0.0003). Regarding individual species within the mixed biofilm, DR9-RR14 led to a 57% reduction in the CFU/mm^2^ of *C. albicans* and a 43% reduction in the CFU/mm^2^ of *S. mutans*.

Lastly, RR14 at 4096 μM also achieved a 21% reduction in mixed biofilm viability (CFU/mm^2^) (*p* = 0.001). When evaluated separately, the viability of *C. albicans* and *S. mutans* was reduced by 62 and 38%, respectively.

### Monitoring Acidogenesis by pH Measurements

The culture medium pH analysis showed a decrease in pH from 7 to 6.5 in the culture medium of the groups treated with His3 and His5 proteins after 48 h, respectively, showing the lowest medians of the analysis (Figure 3c). It was possible to observe the statistical difference among the previous times evaluated: 12, 24, and 36 h. Regarding the results of the peptides DR9-RR14 and RR14, the pH values also decreased over time. RR14 remained higher compared with the groups treated with His3 and His5.

### Quantification of Biofilm Components of Mixed Biofilms

For the dry weight (Figure 4a) of the mixed biofilm, there was no statistical difference among the proteins/peptides evaluated. His3 and His5 reduced the amount of insoluble dry weight by 55 and 57%, respectively (Figure 4b), compared to NaCl (*p* < 0.05). Protein content was also reduced by 63 and 65% for His3 and His5, respectively (Figure 4c). In relation to eDNA (Figure 4d), reductions of 64 and 55% were observed for His3 and His5, respectively. For ASP (Figure 4e), no significant differences were found among the proteins/peptides. Lastly, WSP was reduced by 46 and 32% for His3 and His5 (Figure 4f), respectively, when compared to NaCl (*p* < 0.05). For peptides DR9-RR14 and RR14, results for all ECM components evaluated were statistically similar to the control group (NaCl) (*p* > 0.05).

**Figure 4:**
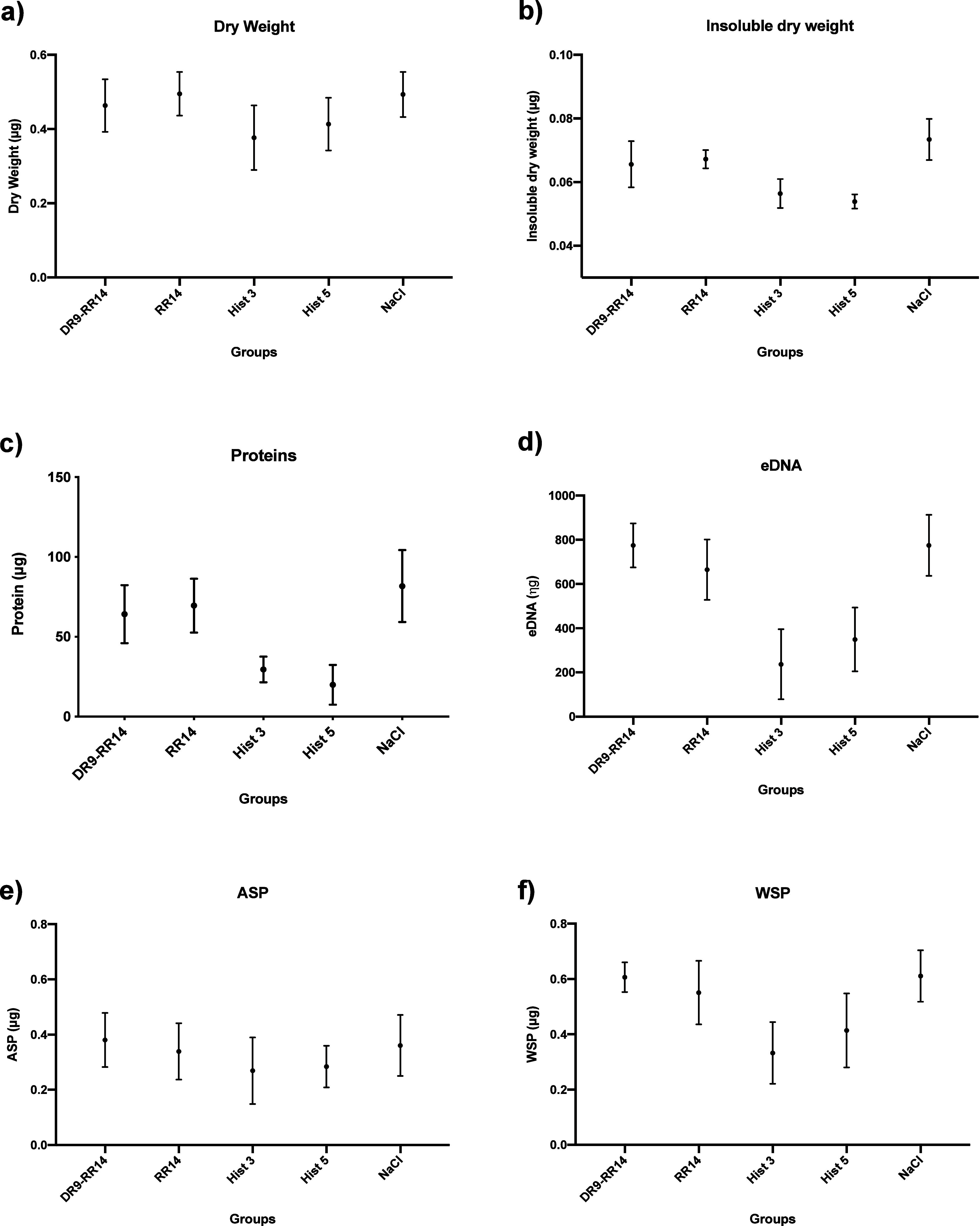
Median and 95% confidence interval of (a) dry weight (μg), (b) insoluble dry weight (μg), (c) proteins (μg), (d) eDNA (ng), (e) ASP (alkali-soluble polysaccharides/μg), and (f) WSP (water-soluble polysaccharides/μg) of mixed biofilm (*C. albicans* and *S. mutans*) formed on acrylic resin against proteins/peptides DR9-RR14 (2048 μM), RR14 (4096 μM), histatin 3 (256 μM), and histatin 5 (512 μM); NaCl: experimental control. Points: data median. Error bars: minimum and maximum values. The nonintersection of error bars denotes statistical difference according to the 95% confidence interval (*p* < 0.05) (*n* = 9/group).

### Structural Analysis of Mixed Biofilms by Confocal Laser Scanning Microscopy (CLSM)

CLSM imaging revealed notable structural differences in the mixed biofilms treated with His3 and His5 compared to those in the NaCl control group (Figure 5). In this group, a strong overlap between green fluorescence (live cells stained with Syto 9) and red fluorescence (dextran-labeled ECM) was observed, indicating dense biofilm formation with intact cell clusters embedded in a robust matrix. In contrast, biofilms treated with His3 and His5 showed visibly reduced green fluorescence, suggesting decreased cell viability, and a more fragmented red signal, indicating disruption of EPS production and matrix integrity.

**Figure 5:**
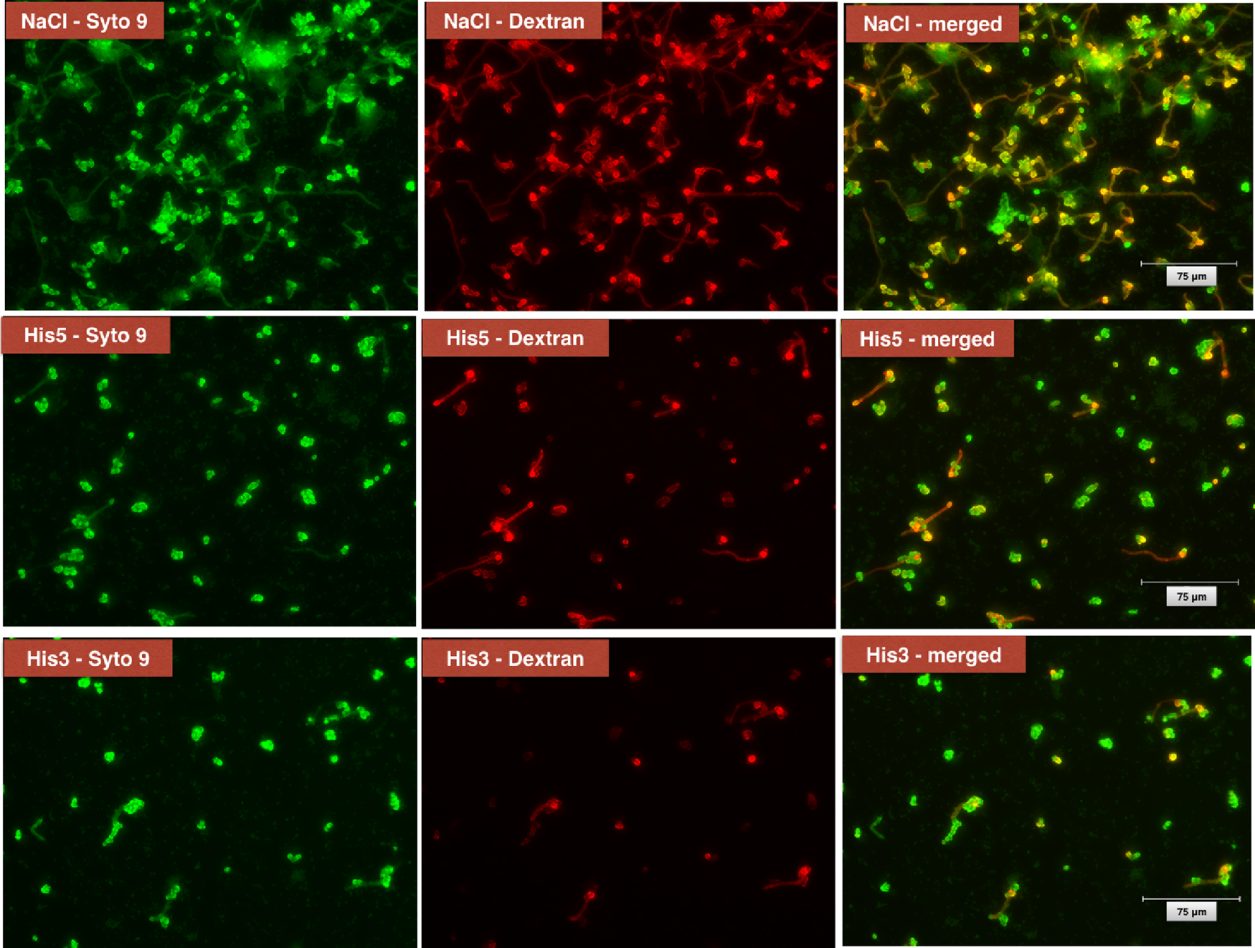
Fluorescence images of the mixed biofilm containing *Candida albicans* and *Streptococcus mutans* after the most promising proteins/peptides were tested in the preliminary tests: histatin 3 (His3) and histatin 5 (His5). Green fluorescence (Syto 9): represents live cells of *C. albicans* and *S. mutans*; red fluorescence (dextran): indicates extracellular matrix (ECM) components surrounding the cells. Merged images: show the colocalization of live cells and the ECM in biofilms treated with NaCl, His5, and His3.

In this present investigation, the merged images revealed weakened colocalization between cells and matrix in both His3 and His5 groups, suggesting reduced biofilm cohesion and ECM structure. These findings align with the CFU/mm^2^ and ECM quantification data, confirming that these proteins/peptides not only reduce biofilm viability but also affect its organization. Additionally, His3-treated biofilms displayed more irregular EPS distribution and signs of spatial breakdown, while His5 treatment led to more isolated cell clusters with a minimal surrounding matrix.

### Morphological Changes in Mixed Biofilms Observed by SEM

SEM was employed to analyze the topography and surface organization of the mixed biofilm after treatments with proteins/peptides (Figure 6). Analysis of the biofilm treated with His5 revealed a lower concentration of microbial cells and a reduced amount of ECM compared to the biofilm treated with His3. Regarding the interspecies dynamic in the His5 group, the images show less interaction compared to His3, with more segregation between species.

**Figure 6:**
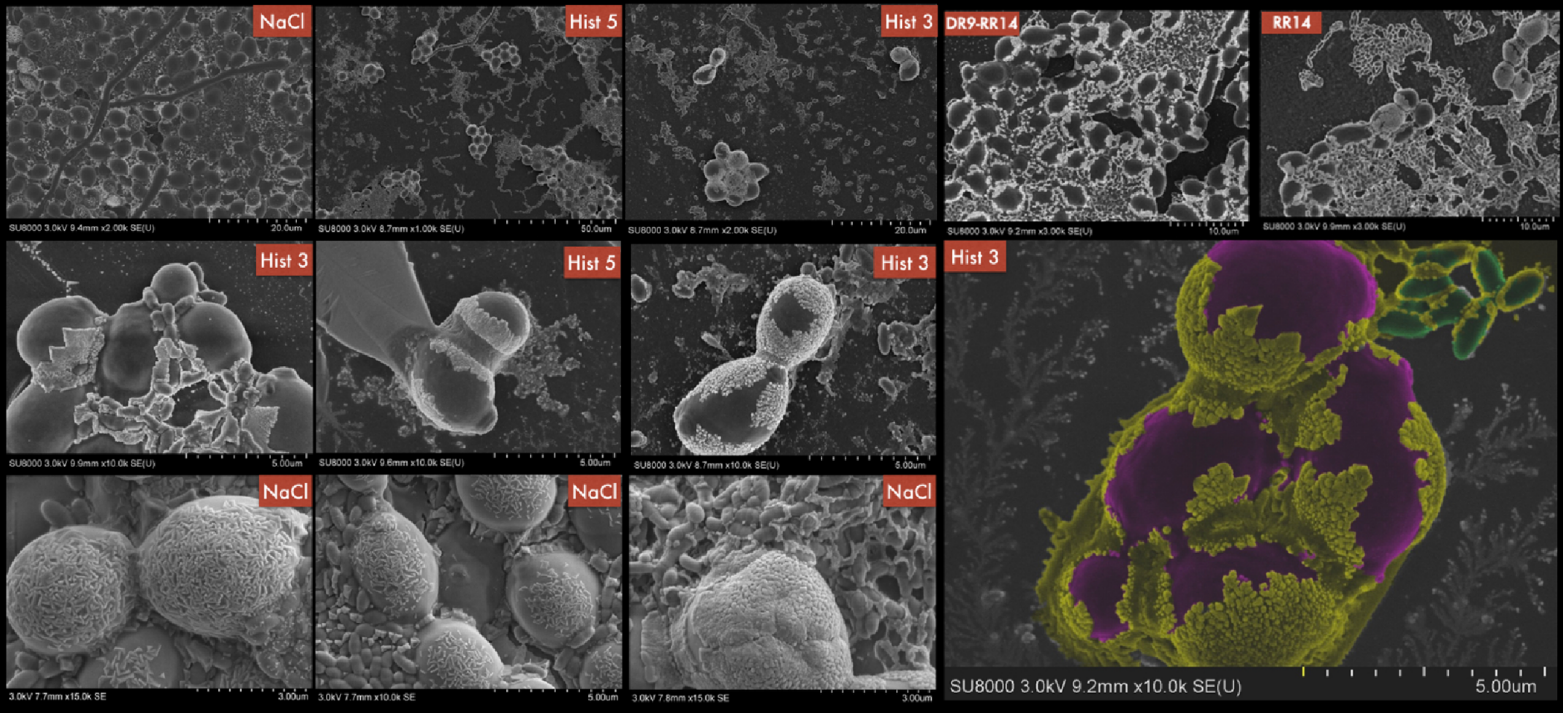
Representative images obtained by SEM of the mixed biofilm*(Candida albicans* and *Streptococcus mutans*) treated with His3, His5, DR9-RR14, and RR14 and control (NaCl). Purple color: *C. albicans*; green color: *S. mutans*; yellow color: extracellular matrix. Magnifications of 5, 10, 15, 20, and 50×. (*n* = 3/group).

The general observation of the mixed biofilm treated with DRR9-RR14 shows gaps and lower cell density compared to NaCl, but still higher cell density than His3 and His5. Both *C. albicans* and *S. mutans* appear to be collapsed with irregular shapes and less defined structures. Regarding the biofilm treated with RR14, it is slightly more disorganized than the NaCl group but less so than the His3 group.

The mixed biofilm of the NaCl group depicts strong interactions between both microorganisms, showing yeast and hyphal forms of *C. albicans* intertwining around clusters of *S. mutans*, suggesting a synergistic relationship between the microorganisms. Additionally, *S. mutans* forms tight clusters surrounded by extracellular material. Moreover, this group exhibits the most complex and structured biofilm among all the groups. The control biofilms treated with NaCl displayed a typical interaction pattern between *C. albicans* and *S. mutans* and reveal a balanced distribution of cells and matrix, suggesting a stable biofilm environment under NaCl conditions.

### Biocompatibility Assessment of Proteins/Peptides by alamarBlue

For DR9-RR14 (Figure 7a), all concentrations tested (1024, 2048, and 4096 μM) behaved statistically similar to CT. The DR9-RR14 concentration of 2048 μM (BIC-2) showed a 19% reduction compared to CT and was also classified as noncytotoxic (<25%) according to ISO guidelines. Regarding RR14 (Figure 7b), the concentration of 4096 μM (BIC-2) reduced cell viability by 22% compared to CT (*p* = 0.07) and was classified as noncytotoxic (<25%) according to ISO guidelines. In addition, all concentrations used in the analysis (2048, 4096, and 8192 μM) were statistically similar.

**Figure 7:**
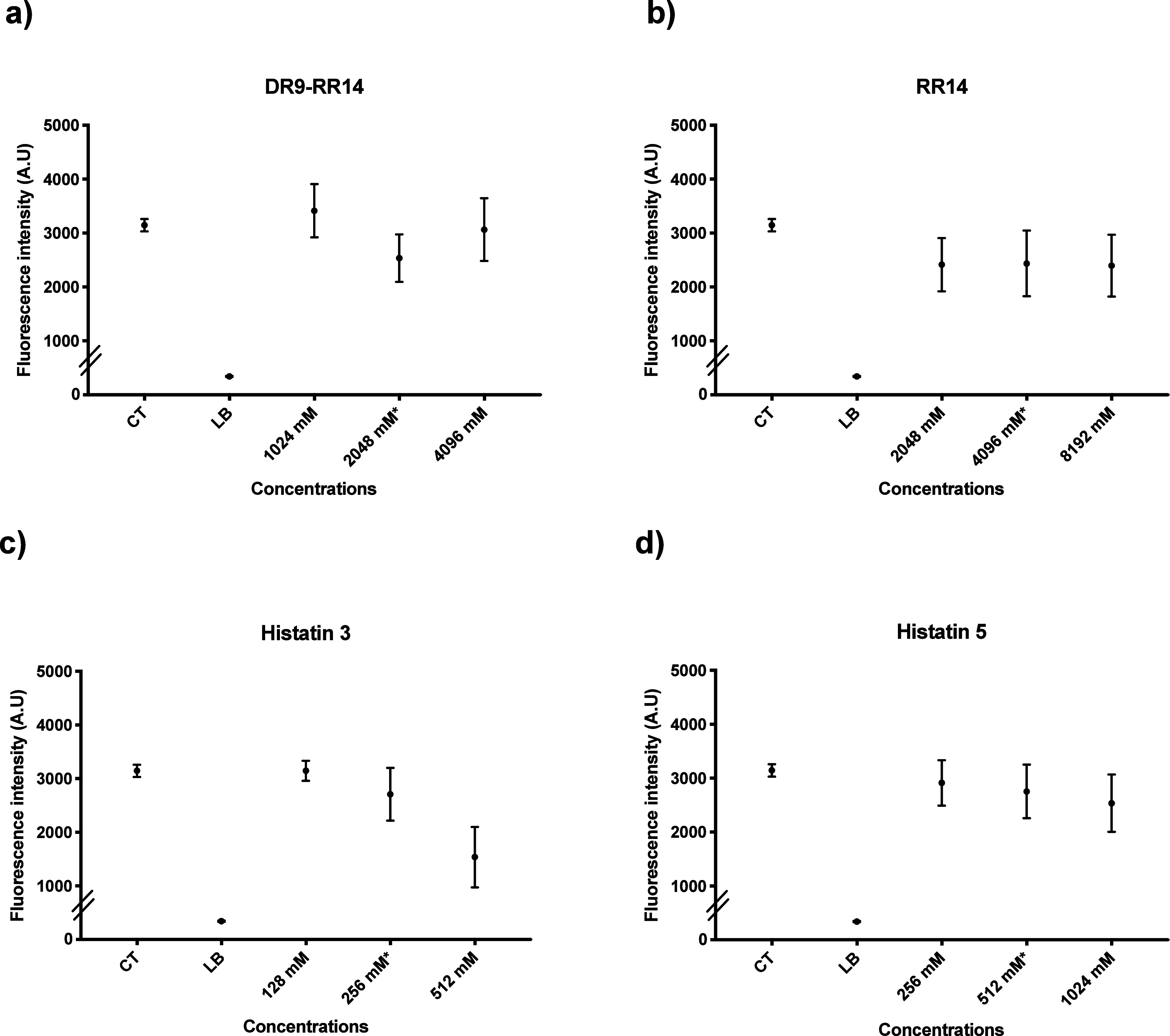
Median and 95% confidence interval of cellular viability through fluorescence intensity (arbitrary units, A.U.) by alamarBlue assay. CT: live cell control; LB: lysis buffer, dead cell control. Points: data medians. Error bars: minimum and maximum values. The nonintersection of error bars denotes statistical difference according to the 95% confidence interval (*n* = 12/group) (*p* < 0.05) (a) DR9-RR14 peptide with 1024 μM, 2048 μM × (BIC-2 concentration) and 4096 μM concentrations. (b) RR14 peptide with 2048 μM, 4096 μM × (BIC-2 concentration) and 8192 μM concentrations. (c) Histatin 3 protein with 128 μM, 256 μM × (BIC-2 concentration) and 512 μM concentrations. (d) Histatin 3 protein with 256 μM, 512 μM × (BIC-2 concentration) and 1024 μM concentrations.

His3 showed statistical similarity (*p* = 0.2) among the concentrations of 128 μM, 256 μM (BIC-2) and CT, with a 13% reduction in viability (Figure 7c). The concentration of 512 μM had the lowest median (2708 A.U.) among the tested concentrations and was statistically different from the others in the analysis (*p* = 0.003). For His5, all concentrations tested (256, 512, and 1024 μM) behaved similarly to CT (Figure 7d), and the BIC-2 concentration (512 μM) showed a 12% reduction compared to that of CT.

## Discussion

The aim of the present study was to investigate the effect of proteins/peptides DR9-RR14, RR14, His3, and His5 against *C. albicans* and *S. mutans* mixed biofilm and their ECM and determine their cytotoxicity on human gingival fibroblasts (FGH), as a means of assessing their suitability for use in controlling denture stomatitis. It has been shown that there is a bacterial–fungal (interkingdom) interaction between these microorganisms in oral microbioma^19^ that can increase the stomatitis pathogenesis.^20^ In this respect, this study was pioneering in quantifying *C. albicans* and *S. mutans* in the same biofilm, as well as the biofilm composition adhered on acrylic resin after the protein/peptide pellicle formation with DR9-RR14, RR14, His3, and His5.

Among all proteins/peptides tested, the results showed that His3 had the lowest BIC-2 at 256 μM, with 57% absorbance reduction compared to the GC and 59% reduction in colony-forming units (CFU/mL) compared to the GC. These results support findings from a previous study in which authors screened the minimum inhibitory concentration (MIC) of His3 against *S. mutans* U159 planktonic culture (single-species) by absorbance (620 nm) and found that 128 μM concentration had the highest antimicrobial efficacy compared to other proteins/peptides (statherin, DR9, DR/2, DR9-DR9, RR14, histatin 1, and histatin 5).^21^ It is understood that the MIC (128 μM) found in *S. mutans* planktonic cultures (single-species) is lower than the BIC-2 of mixed biofilm because there is a cooperative relationship with bacterium–fungal association.^21–23^ Both microorganisms provide metabolites and/or substrates that stimulate each other and prolong mixed biofilm survival, making this model more difficult to inactivate than planktonic cultures containing a single species.^6^

Regarding the viability of cells of mixed biofilms, His3 (256 μM) was statistically significant (*p* = 0.023), with 46% reduction in mixed biofilm compared to the GC. His5 (512 μM) also showed antibiofilm activity with a reduction of 41% compared to the GC, which was statistically significant (*p* = 0.038). Previous studies have shown that His5 is already effective in reducing the metabolic activity of *C. albicans* in single species by 50% at a lower concentration (4.2 μM) than was found in this study (512 μM).^24^ This result can be explained by the fact that polymicrobial biofilms have more extracellular components in their ECM than single biofilms;^19^ it can be more difficult to avoid cell adhesion by the microorganisms and require higher concentrations to affect this multispecies biofilm. This hypothesis was supported by our findings, since there was greater susceptibility of the *C. albicans* and *S. mutans* treated with proteins/peptides and quantified within mixed biofilm, when compared to the overall mixed biofilm. However, His3 and His5 also promoted a slight reduction in the pH of the biofilm (7 to 6.54—SD ± 0.04) after 48 h of formation, meaning that, despite the decrease in cell viability found here, it was not enough to increase acidogenicity. The mixed biofilm treated with DR9-RR14 and RR14 peptides showed the highest means of the analysis (1.29 × 10^04^ and 1.07 × 10^04^, respectively) with reduction of approximated 20% with both peptides, when compared to NaCl.

The EPS matrix provides mechanically stable and complex chemical microenvironments that are fundamental for protection/scaffolding of biofilms.^25–27^ Due to this, in-depth investigation of EPS matrix-mixed communities, particularly in polymicrobial biofilm models, is necessary to establish the optimal concentration required to avoid the adhesion and formation of this microenvironment.^28^ In the present investigation, His3 and His5 are considered the most efficient proteins, which caused distinct reduction in insoluble dry weight, protein, eDNA, and WSP when compared to the NaCl group. Conversely, no statistically significant differences (*p* > 0.05) were revealed between the total biomass (dry weight) and ASP among all proteins/peptides evaluated. These results show that His3 and His5 may affect the structural integrity of the biofilm (eDNA) and interfere with the enzymatic hydrolysis of polysaccharides (WSP).^28,29^ Additionally, the reduction in proteins found here also indicate that both His3 and His5 show promise since they appear to play an important role in the biofilm’s dynamic, acting as a digestive microstructure that ruptures extracellular biopolymers to obtain energy.^20^ While biofilms will still be formed, this study shows that treating the substrate (acrylic resin) with proteins/peptides can control cell colonization on the surface and avoid the maturation of this ECM.

SEM and fluorescence analyses provided a detailed visualization of the structural differences^30^ and interactions within mixed biofilms of *C. albicans* and *S. mutans*, treated with proteins/peptides. A key observation from SEM was finding less ECM in the biofilms treated with His3 and His5 compared to DR9-RR14, RR14, and NaCl, which supports our CFU/mm^2^ results. In addition, the morphological polymorphism of *C. albicans* (yeast and hypha forms) was noticed in the His3 group, likely indicating a dynamic response to the treatment, which improves biofilm resilience. Moreover, SEM images show reduced interaction, followed by increase of segregation between *C. albicans* and *S. mutans* in the His5 group, showing a less synergistic interaction, which might influence the biofilm’s susceptibility to antimicrobial interventions. According to analysis of the fluorescence images acquired by CLSM, His5 and His3 treatments have different impacts on biofilm structure and dynamics; His5 appears to reduce the protective matrix, likely causing susceptibility to face the treatment for the biofilm. Conversely, His3 promotes intertwining in the biofilm architecture, which might protect against both physical and pharmacological challenges.^17^

In addition to Histatins inhibiting mixed biofilm of *C. albicans* and *S. mutans*, the peptides used here are also biocompatible with FGH in the monolayer. All proteins/peptides were classified as noncytotoxic (<25%) from ISO^31^, enabling the use of its in the oral environment without damaging the healthy oral cells.^32^ His3, at a concentration of 256 μM (BiC-2), showed 13% viability reduction displaying statistical similarity with the CT group. Moreover, the same protein at a concentration of 512 μM displayed a significant difference in the analysis. Regarding His5, at a concentration of 512 μM (BiC-2), AMP displayed 12% reduction in cell viability in comparison to the growth control. In addition, the concentrations of His5 evaluated in this study (256, 512, and 1024 μM) were similar statistically. The concentrations studied here displayed biocompatibility to FGH, similar to a previous in vitro study, which tested His5 concentrations from 800 to 6.25 μg/mL also on human cells.^33^ The concentration used here for BIC-2 (512 μM) is within the range established by these authors.

## Conclusions

Among the proteins/peptides evaluated, His3 demonstrated the most promising results in preventing adhesion and colonization of *C. albicans* and *S. mutans* resistant biofilms on acrylic resin surfaces, followed by His5. They significantly reduced cell viability (CFU/mm^2^) and biomass (insoluble dry weight) while also disrupting vital constituents of the ECM (proteins, eDNA, and WSP). All of the proteins/peptidestested here were also biocompatible with FGH cells. Given the role *C. albicans* and *S. mutans* play in stomatitis, these in vitro results show that His3 and His5 may play a role in potential therapeutics for denture stomatitis, with further investigation using in vivo models and subsequent clinical trials required.

## Materials and Methods

### Strains and Cell Culture Condition

The strains *S. mutans* UA159 and *C. albicans* ATCC 10231 (fluconazole-resistant) were used to prepare mixed biofilms. Each strain was grown on Brain Heart Infusion (BHI) agar plates (48 h/37 °C/5% CO_2_). Five to ten colonies of each microorganism were inoculated into 10 mL of culture medium composed of tryptone with yeast extract containing 1% of glucose (TYE+1% glucose; tryptone: 10 g/L, yeast extract: 5 g/L, glucose: 10 g/L) and incubated (37 °C/5% CO_2_). After 16 h, 1:20 dilutions of each starter culture were performed in TYE + 1% glucose medium, and the cultures were grown until the mid log growth phase (OD 600 nm: 0.74 ± 0.03 adjusted in 1.0 × 10^7^ CFU/mL for *C. albicans* and 0.63 ± 0.08 adjusted in 1.0 × 10^8^ CFU/mL for *S. mutans*).

### Biofilm Inhibitory Concentration (BIC-2)

For the BIC-2 assay, the Clinical and Laboratory Standards Institute (CLSI)^34^ broth microdilution method was used as a reference.^35^ First, to standardize the population, both strains (inoculum) were diluted to the same concentration (CLSI parameters: 1 × 10^3^) and 50 μL of each microorganism were incubated in 96-well plates with 100 μL of culture medium (TYE+1% glucose) for 8 h (biofilm adhesion phase). Next, the wells were washed three times, and the proteins/peptides DR9-RR14, RR14, His 3, and His 5 were dissolved in NaCl (pH 7.4) in dilutions ranging from 0 to 1024 μM. As a contamination control (CC; NaCl), 100 μL of TYE and 100 μL of NaCl (without cells and proteins/peptides) were added to the well to ensure that any observed effects were not due to contaminants in the medium or reagents. As a GC, only mixed biofilm (100 μL of TYE + 100 μL of NaCl) was tested without protein/peptide (drug-free control). The 96-well plates were incubated at 37 °C and visually observed for the presence or absence of biofilm growth after 24 h (BIC-2). Each well was scraped with a tip for 45 s to detach the adhered cells from the bottom of the plate to obtain a biofilm suspension, followed by plating to obtain the minimum concentration of each peptide necessary to inhibit a twofold biofilm formation. The BIC-2 value is the concentration of the proteins/peptides required to inhibit 50% of biofilm formation, which was compared to the control treatment (GC).^35^

### Acrylic Resin Disc Standardization and Protein/Peptide Pellicle Formation

Acrylic resin (VIPI Dental Products, Pirassununga, São Paulo, Brazil) discs with roughness between 2.7 and 3.7 μm (Ra) were obtained from the cutter (dimensions: 7 mm length, 5 mm width, and 2 mm thickness) and positioned vertically in the acrylic holder of the lid assembly. The assembled lid with the acrylic discs and the culture plate were sterilized by exposing them to ultraviolet light for 1 h.^15^ Acrylic holders with a 2 mm-depth slot were bonded to the center of the lid of a 96-well plate using wax to secure the samples. The total surface area of each slab was measured to standardize CFU colonies, ensuring accurate comparisons of biofilm density across samples and be secure to apply the CFU/mm^2^ formula. They were distributed randomly to each of the following treatment groups (200 μL/well/slab): DR9-RR14 (2048 μM), RR14 (1024 μM), His3 (256 μM), His5 (512 μM), or NaCl (pH 7.2). The acrylic discs with the proteins/peptides were incubated for 2 h at 37 °C under constant agitation at 60 rpm/min, corresponding to protein/peptide pellicle formation.^18^

### *C. albicans* and *S. mutans* Mixed Biofilm Formation

After the protein/peptide pellicle formation, the lids with the acrylic resin discs were washed 3× with 5 mM NaCl pH 6.8 and then transferred to 24-well culture well plates having the standard cell suspensions (inoculum) (*C. albicans* at 1.0 × 10^7^ CFU/mL and *S. mutans* at 1.0 × 10^8^ CFU/mL)^15^ with culture medium (TYE + 1% glucose). The biofilms were allowed to adhere for 8 h at 37 °C and 10% CO_2_ (biofilm adhesion phase).^36^ As contamination control (CC), wells received 1 mL of TYE and 1 mL of NaCl (without microorganisms and proteins/peptides). As a GC, only mixed biofilm (1 mL of TYE + 1 mL of NaCl) was tested without protein/peptide (drug-free control). After the adhesion phase (8 h), the acrylic resins bonded on the lid were transferred to fresh TYE medium supplemented with 0.1 mM glucose to induce initial biofilm maturation and rested overnight (16 h) at 37 °C and 10% CO_2_. The discs were washed three times with PBS and then transferred to a new 96-well plate containing fresh TYE medium supplemented with 1% sucrose, which promotes enhanced extracellular polysaccharide production necessary for mature biofilm architecture and stability.^15^ This process was repeated at regular intervals, until the biofilm formation reached 48 h (Figure 8a).

**Figure 8:**
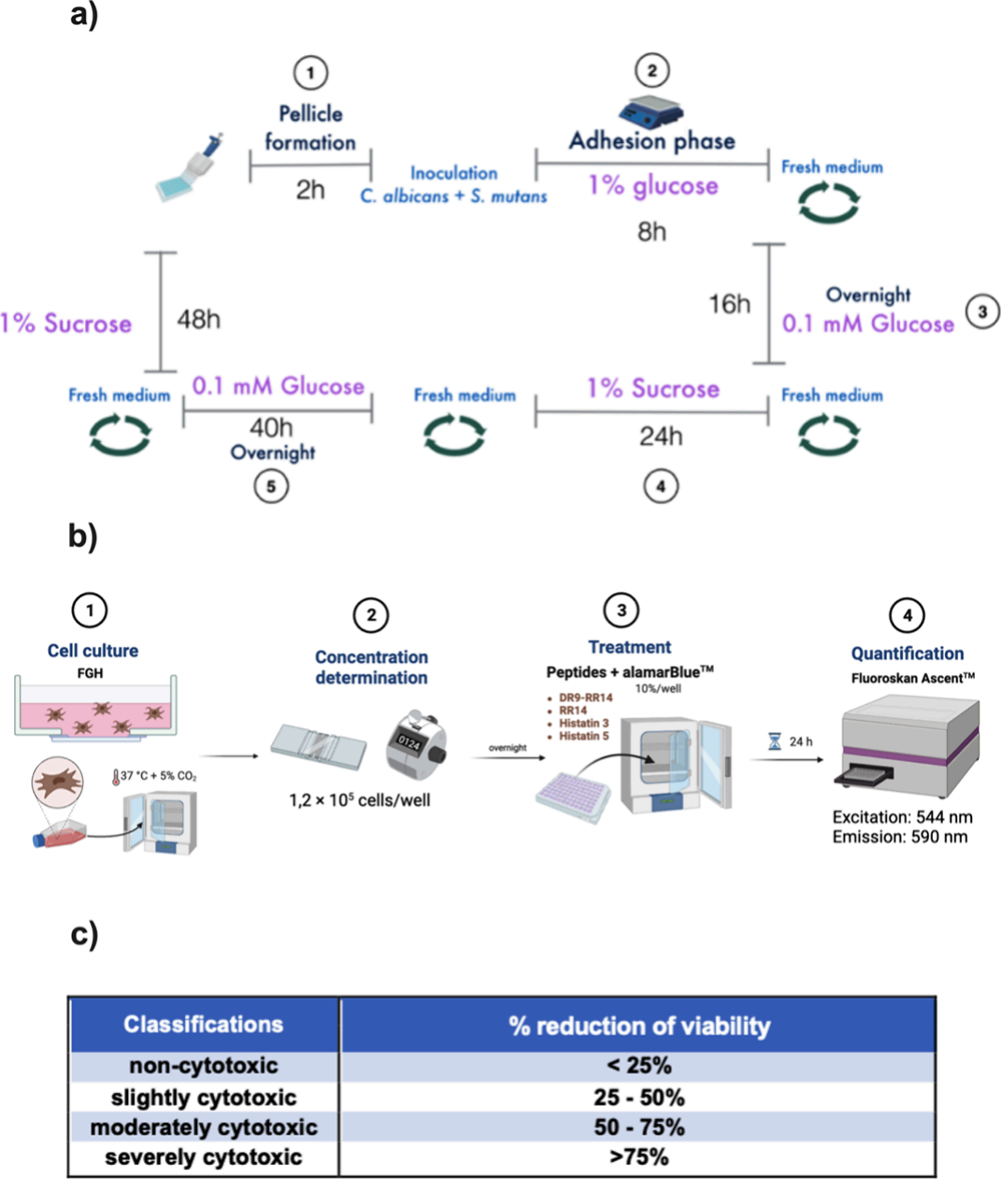
(a) Flowchart depicting the development of mixed biofilm (*Streptococcus mutans* UA159 and *Candida albicans* ATCC 10231) on acrylic resin treated with protein/peptide. (b) Flowchart of alamarBlue assay. Classification of cytotoxicity according to the cell viability reduction after treatment compared to control by ISO 10993-5:2009 guidelines (2020). Created in BioRender. Lab, S. (2025) https://BioRender.com/a33w751.

### Culture Medium pH Analyses

Throughout the 48 h biofilm development period, the pH of the culture medium was measured both before and after each medium replacement at the specific time points (0, 12, 24, 36, and 48 h). These measurements were performed to evaluate changes in the pH and acidogenesis of the developing biofilm over time. The pH meter was calibrated (pH between 4.0 and 7.0 standards), and a pH electrode was used to measure the pH of an aliquot from each well.

### Determination of Cell Viability Biofilm Formed on Acrylic Resin (CFU/mm^2^)

Immediately after 48 h of biofilm development, the wells were washed 3× (0.9% NaCl) and 100 μL aliquots of the biofilm culture from each well were serially diluted (10^–4^ to 10^–7^) in NaCl (pH 6.8). Subsequently, samples were plated in duplicate 20 μL in Petri dishes containing BHI agar. All plates were incubated aerobically at 37 °C for 48 h with aqueous CO_2_. Next, viable colonies were counted, and the number of colony-forming units (CFU) was divided per mm^2^ of each slab (previously measured) to obtain total area as the area of the discs previously measurement (CFU/mm^2^) (*n* = 9/group).^15^

### Quantification of Biofilm Components of Mixed Biofilms

After the CFU/mm^2^ was determined by plating the biofilm, the remaining volume from each sample was aliquoted and processed for the quantification of individual ECM components. The same biofilm samples were used across both assays, ensuring sample size consistent (*n* = 9/group).^37^

Specifically, 100 μL was used for dry weight and insoluble dry-weight quantification. To assess protein content, 150 μL aliquots were prepared for analysis using a BCA standard curve with bovine serum albumin with quantification performed by spectrophotometry (optical density: OD_560nm_).^38^ For extracellular DNA (eDNA), 650 μL was extracted via phenol:chloroform:isoamyl alcohol and analysis by NanoDrop (OD_260nm_).^6^ Water-soluble polysaccharides (WSP) were evaluated using 1 mL aliquots through ethanol precipitation followed by phenol–sulfuric acid detection (OD_490nm_).^39^ For alkali-soluble polysaccharides (ASP), 950 μL of the resuspended pellet was processed by using the same WSP colorimetric assay after drying.

### Scanning Electron Microscopy (SEM)

New mixed biofilm samples were grown according to the previously described protocol, allowing biofilm development over 48 h. After all the development process, the acrylic resin discs were then washed (0.89% NaCl) to remove nonadherent cells and underwent a multistep dehydration process using an ethanol series: 70% ethanol for 60 min, 90% ethanol for 60 min, followed by five 30 min washes with absolute ethanol. Next, the acrylic resin discs were dried in a desiccator with silica for 7 days. For analysis, samples were mounted on aluminum stubs, sputter-coated with gold, and examined using a JEOL JSM 6610LV SEM at magnifications of 2000×, 3000×, and 5000×. This method allowed for a detailed visualization of the biofilm structure at various levels of magnification (*n* = 3/group).

### Fluorescence Images by Confocal Laser Scanning Microscopy (CLSM)

A staining methodology was employed to better visualize the overall microbial architecture and viability for the mixed biofilms with*C. albicans* and *S. mutans*. The acrylic resin discs treated with protein/peptide and the mixed biofilm grown were submitted to biofilm formations as previously described. After the period of adhesion (8 h), the medium was replaced with fresh medium, and 13.4 μL of 1 mM Alexa Fluor 647 fluorophore-labeled dextran was added in the wells. Biofilm development continued as previously described, allowing the dextran to incorporate into the growing ECM during biofilm development. After the biofilm maturation period (19 h; 37 °C; 10% CO_2_),^40^ the samples were gently washed (3×; PBS) to remove loosely attached cells. 1.5 μL of SYTO9 (in PBS at 6.7 μM) was placed into the well for 30 min, to stain the acid nucleus of the cells. After staining, the biofilms were washed (3×; PBS) and images were acquired using CLSM (CARL ZEISS LSM 800; Airyscan GaAsp detector, Germany) to visualize the ECM (the pre-incorporated dextran) and the nucleus of microbial cells (Syto 9) within the biofilm.

### Cytotoxicity Evaluation of Peptides/Proteins on Fibroblast Cells by alamarBlue

The FGH (human gingival fibroblasts) cells were thawed and cultured in Dulbecco’s Modified Eagle Medium (DMEM; GIBCO, Grand Island, New York, USA) with high glucose (4.5 g/L), supplemented with 2.0 mmol L^–1^ of glutamine (Lonza, Basel, 10% of bovine serum), SFB (Gibco, Grand Island, New York, USA), and 1% antibiotic/antimycotic (penicillin G 10,000 μg·mL^–1^, streptomycin 10,000 μg·mL^–1^, amphotericin B 25 μg·mL^–1^) (Sigma-Aldide, St. Louis, Missouri) and kept in the incubator, with 5% CO_2_. The cells were repeated until reaching 80% confluence, and the bottle was washed (3×) with PBS (pH 7.2). Next, the cells were counted and plated at 1.2 × 10^5^ cells/well, between the third and eighth passages. The cells were then resuspended to a volume of 200 μL of cells in DMEM and added to 96-well plates at the amount established before for each cell line, to be incubated at 37 °C and 5% CO_2_ for 16 h. Afterward, the wells were washed (3×; PBS) and the protein/peptide concentrations were adjusted, according to the optimal breakpoint obtained previously, followed by 24 h of exposure (based on BIC-2) (200 μL/well): DR9-RR14 (2048 μM), RR14 (1024 μM), His3 (256 μM), and His5 (512 μM) or NaCl (pH 7.2). Furthermore, cells were also exposed to four additional protein/peptide concentrations (two concentrations higher and two concentrations lower) based on the ranking established previously. For the cell viability quantification, 10% of alamarBlue with 90% of DMEM supplemented with FBS was added, incubated for 24 h, and measured with Synergy BioTek by end point (FL, Thermo Scientific; excitation, 544 nm; emission, 590 nm) (Figure 8b). As live control (CT), cells were included without any treatment. As death control (lysis buffer), Triton X-100 (0.9%) was used The results obtained were normalized and classified with respect to cytotoxicity according to ISO 10993-5:2009 guidelines (Figure 8 c).^31^

## Statistical Analysis

To assess the distribution and homogeneity of the data, Shapiro–Wilk and Levene tests were applied. Since the data of BIC-2, CFU/mm^2^ and biofilm components did not show a normal distribution; the analysis was performed with Kruskal–Wallis test with Dunn post hoc. The data of alamarBlue showed normal distribution and homogeneity of the variances, and one-way analysis of variance test was performed followed by Tukey post hoc test. All the statistical calculations were performed using IBM SPSS 30.0.0 version. Additionally, the graphs were created with GraphPad Prism version 5.0. A *p*-value of less than 0.05 (*p* < 0.05) was considered statistically significant.

## References

[1] Scully C., EI-Kabir M., Samaranayake L. P. (1994). Candida and Oral Candidosis: A Review. Critical Reviews in Oral Biology & Medicine.

[2] Ishikawa K. H., Mayer M. P. A., Miyazima T. Y., Matsubara V. H., Silva E. G., Paula C. R., Campos T. T., Nakamae A. E. M. (2015). A Multispecies
Probiotic Reduces Oral *Candida* Colonization in Denture
Wearers. Journal of Prosthodontics.

[3] Thein Z. M., Samaranayake Y. H., Samaranayake L. P. (2007). Characteristics of Dual Species Candida
Biofilms on Denture Acrylic Surfaces. Arch Oral
Biol..

[4] Hwang G., Marsh G., Gao L., Waugh R., Koo H. (2015). Binding Force
Dynamics of Streptococcus Mutans-Glucosyltransferase B to Candida
Albicans. J. Dent Res..

[5] Karatan E., Watnick P. (2009). Signals, Regulatory
Networks, and Materials That Build
and Break Bacterial Biofilms. Microbiol Mol.
Biol. Rev..

[6] Panariello B. H. D., Klein M. I., Dias L. M., Bellini A., Costa V. B., Barbugli P. A., Pavarina A. C. (2021). Lactobacillus Casei Reduces the Extracellular
Matrix Components of Fluconazole-Susceptible Candida Albicans Biofilms. Biofouling.

[7] Gad M. M., Fouda S. M. (2020). Current Perspectives
and the Future of Candida Albicans-Associated
Denture Stomatitis Treatment. Dent Med. Probl.

[8] Boparai J.
K., Sharma P. K. (2019). Mini Review
on Antimicrobial Peptides, Sources, Mechanism
and Recent Applications. Protein Pept Lett..

[9] Bechinger B., Gorr S.-U. (2017). Antimicrobial Peptides: Mechanisms of Action and Resistance. J. Dent Res..

[10] Siqueira W. L., Zhang W., Helmerhorst E. J., Gygi S. P., Oppenheim F. G. (2007). Identification
of Protein Components in in Vivo Human Acquired Enamel pellicle Using
LC-ESI-MS/MS. J. Proteome Res..

[11] Siqueira W. L., Margolis H. C., Helmerhorst E. J., Mendes F. M., Oppenheim F. G. (2010). Evidence
of Intact histatins in the in Vivo Acquired Enamel pellicle. J. Dent Res..

[12] Siqueira W. L., Helmerhorst E. J., Zhang W., Salih E., Oppenheim F. G. (2007). Acquired
Enamel pellicle and Its Potential Role in Oral Diagnostics. Ann. N.Y. Acad. Sci..

[13] Hojo K., Nagaoka S., Ohshima T., Maeda N. (2009). Bacterial Interactions
in Dental Biofilm Development. J. Dent Res..

[14] Komatsu T., Salih E., Helmerhorst E. J., Offner G. D., Oppenheim F. G. (2011). Influence
of histatin 5 on Candida Albicans Mitochondrial Protein Expression
Assessed by Quantitative Mass Spectrometry. J. Proteome Res..

[15] Marin L. M., Xiao Y., Cury J. A., Siqueira W. L. (2022). Modulation of Streptococcus
Mutans Adherence to Hydroxyapatite by Engineered Salivary Peptides. Microorganisms.

[16] Basiri T., Johnson N. D., Moffa E. B., Mulyar Y., Serra Nunes P. L., Machado M. A. A. M., Siqueira W. L. (2017). Duplicated or Hybridized Peptide
Functional Domains Promote Oral Homeostasis. J. Dent Res..

[17] Valente M. T., Moffa E. B., Crosara K. T. B., Xiao Y., de Oliveira T. M., Machado M. A. de A. M., Siqueira W. L. (2018). Acquired Enamel
pellicle Engineered
Peptides: Effects on Hydroxyapatite Crystal Growth. Sci. Rep.

[18] Pellissari C. V. G., Jorge J. H., Marin L. M., Sabino-Silva R., Siqueira W. L. (2021). Statherin-Derived Peptides as Antifungal Strategy against
Candida Albicans. Arch Oral Biol..

[19] Kim D., Sengupta A., Niepa T. H. R., Lee B.-H., Weljie A., Freitas-Blanco V. S., Murata R. M., Stebe K. J., Lee D., Koo H. (2017). Candida Albicans
Stimulates Streptococcus Mutans Microcolony Development
via Cross-Kingdom Biofilm-Derived Metabolites. Sci. Rep.

[20] Pereira-Cenci T., Deng D. M., Kraneveld E. A., Manders E. M. M., Del
Bel Cury A. A., ten Cate J. M., Crielaard W. (2008). The Effect
of Streptococcus Mutans and Candida Glabrata on Candida Albicans Biofilms
Formed on Different Surfaces. Arch Oral Biol..

[21] Moussa D. G., Siqueira W. L. (2021). Bioinspired Caries Preventive Strategy via Customizable
Pellicles of Saliva-Derived Protein/Peptide Constructs. Sci. Rep.

[22] Rocha G. R., Florez Salamanca E. J., de Barros A. L., Lobo C. I. V., Klein M. I. (2018). Effect
of Tt-Farnesol and Myricetin on in Vitro Biofilm Formed by Streptococcus
Mutans and Candida Albicans. BMC Complement
Altern Med..

[23] Lobo C. I. V., Rinaldi T. B., Christiano C. M. S., De Sales Leite L., Barbugli P. A., Klein M. I. (2019). Dual-Species Biofilms of *Streptococcus Mutans* and *Candida Albicans* Exhibit More Biomass and Are Mutually Beneficial Compared with Single-Species
Biofilms. J. Oral Microbiol.

[24] Konopka K., Dorocka-Bobkowska B., Gebremedhin S., Düzgüneş N. (2010). Susceptibility
of Candida Biofilms to histatin 5 and Fluconazole. Antonie Van Leeuwenhoek.

[25] Flemming H.-C., Neu T. R., Wozniak D. J. (2007). The EPS
Matrix: The “House
of Biofilm Cells”. J. Bacteriol..

[26] Nett J. E., Andes D. R. (2020). Contributions of the Biofilm Matrix to Candida Pathogenesis. J. Fungi.

[27] Nett J., Lincoln L., Marchillo K., Massey R., Holoyda K., Hoff B., VanHandel M., Andes D. (2007). Putative Role of Beta-1,3
Glucans in Candida Albicans Biofilm Resistance. Antimicrob. Agents Chemother..

[28] Bowen W. H., Burne R. A., Wu H., Koo H. (2018). Oral Biofilms: Pathogens,
Matrix, and Polymicrobial Interactions in Microenvironments. Trends Microbiol.

[29] Sims K. R., Maceren J. P., Liu Y., Rocha G. R., Koo H., Benoit D. S. W. (2020). Dual Antibacterial
Drug-Loaded Nanoparticles Synergistically
Improve Treatment of Streptococcus Mutans Biofilms. Acta Biomater.

[30] Panariello B. H. D., Klein M. I., Mima E. G. D. O., Pavarina A. C. (2018). Fluconazole Impacts
the Extracellular Matrix of Fluconazole-Susceptible and -Resistant *Candida Albicans* and *Candida Glabrata* Biofilms. J. Oral Microbiol.

[31] International Organization for Standardization (ISO) 23. ISO 10993–5:2009 (Biological evaluation of medical devices – part 5: tests for in vitrocytotoxicity). https://www.iso.org/standard/36406.html. https://www.iso.org/standard/36406.html (accessed 2023–11–18).

[32] da
Silva Pimentel B. N. A., Marin-Dett F. H., Assis M., Barbugli P. A., Longo E., Vergani C. E. (2022). Antifungal Activity and Biocompatibility
of α-AgVO3, α-Ag2WO4, and β-Ag2MoO4 Using a Three-Dimensional
Coculture Model of the Oral Mucosa. Front. Bioeng.
Biotechnol..

[33] Moffa E. B., Machado M. A. A. M., Mussi M. C. M., Xiao Y., Garrido S. S., Giampaolo E. T., Siqueira W. L. (2015). In Vitro Identification of histatin
5 Salivary Complexes. PLoS One.

[34] Wiederhold, N. P. Antifungal Susceptibility of Yeasts and Filamentous Fungi by CLSI Broth Microdilution Testing. In Antifungal Drug Resistance: Methods and Protocols; Humana: New York, NY, 2023; pp 3–16. 10.1007/978-1-0716-3155-3_1. 37024691

[35] Van
Dijck P., Sjollema J., Camue B. P. A., Lagrou K., Berman J., d’Enfert C., Andes D. R., Arendrup M. C., Brakhage A. A., Calderone R., Cantón E., Coenye T., Cos P., Cowen L. E., Edgerton M., Espinel-Ingroff A., Filler S. G., Ghannoum M., Gow N. A.R., Haas H., Jabra-Rizk M. A., Johnson E. M., Lockhart S. R., Lopez-Ribot J. L., Maertens J., Munro C. A., Nett J. E., Nobile C. J., Pfaller M. A., Ramage G., Sanglard D., Sanguinetti M., Spriet I., Verweij P. E., Warris A., Wauters J., Yeaman M. R., Zaat S. A. J., Thevissen K. (2018). Methodologies
for in Vitro and in Vivo Evaluation of Efficacy of Antifungal and
Antibiofilm Agents and Surface Coatings against Fungal Biofilms. Microb. Cell.

[36] Tasso C. O., Ribeiro Ribas B., Morandin Ferrisse T., Silva de Oliveira J., Jorge J. H. (2024). The Antimicrobial Activity of an Antiseptic Soap against
Candida Albicans and Streptococcus Mutans Single and Dual-Species
Biofilms on Denture Base and Reline Acrylic Resins. PLoS One.

[37] Garcia B. A., Panariello B. H. D., Freitas-Pontes K. M.
de, Duarte S. (2021). Candida Biofilm
Matrix as a Resistance Mechanism against Photodynamic Therapy. Photodiagnosis Photodyn Ther.

[38] Lin Q., Zheng Y., Wang G., Shi X., Zhang T., Yu J., Sun J. (2015). Protein Adsorption
Behaviors of Carboxymethylated Bacterial
Cellulose Membranes. Int. J. Biol. Macromol..

[39] DUBOIS M., GILLES K., HAMILTON J. K., REBERS P. A., SMITH F. (1951). A Colorimetric
Method for the Determination of Sugars. Nature.

[40] Koo H., Xiao J., Klein M. I., Jeon J. G. (2010). Exopolysaccharides
Produced by Streptococcus Mutans Glucosyltransferases Modulate the
Establishment of Microcolonies within Multispecies Biofilms. J. Bacteriol..

